# Personalized bleeding management in total knee arthroplasty using reference change value: comparative analysis of tourniquet versus non-tourniquet techniques

**DOI:** 10.1007/s00402-025-05946-1

**Published:** 2025-06-07

**Authors:** Fatih Ugur, Mehmet Akif Bildirici, Recep Taskin, Bedrettin Akar, Mehmet Albayrak, Engin Karadeniz

**Affiliations:** 1 Kastamonu University Faculty of Medicine, Department of Orthopaedics and Traumatology, Kastamonu, Turkey; 2 Kastamonu University Faculty of Medicine, Department of Biochemistry, Kastamonu, Turkey; 3 Sakarya Yenikent State Hospital, Department of Orthopaedics and Traumatology, Sakarya, Turkey; 4 Rumeli University Vocational School of Health Services, İstanbul, Turkey; 5 Kastamonu Education and Research Hospital Orthopaedics and Traumatology Department, Kastamonu, Turkey

**Keywords:** Total knee arthroplasty, Perioperative blood loss, Reference change value, Tourniquet, Individualized hemostasis assessment

## Abstract

**Introduction:**

Total knee arthroplasty (TKA) is a commonly performed surgical procedure for the treatment of advanced-stage knee osteoarthritis. This study aims to compare perioperative blood loss between tourniquet-assisted and tourniquet-free TKA using the reference change value (RCV), a personalized analytical tool that accounts for both biological and analytical variability.

**Materials and methods:**

A retrospective analysis was conducted on 137 patients (tourniquet group (*n* = 68) and non-tourniquet group (*n* = 69) who underwent primary TKA. Hematological parameters—including red blood cell count (RBC), hemoglobin (HGB), and hematocrit (HCT)—were evaluated preoperatively, on the day of surgery, and on postoperative day one. Blood loss was assessed using both conventional statistical methods and RCV-based analysis.

**Results:**

No statistically significant differences were observed between the two groups in terms of age and gender (*p* > 0.05). Although preoperative hemoglobin levels were significantly different between groups (*p* < 0.05), there were no significant intergroup differences in pre- or postoperative RBC and HCT values. Traditional statistical analysis showed no significant difference in blood loss (*p* > 0.05). However, RCV analysis revealed that while bleeding had stabilized by the first postoperative day in the non-tourniquet group, it remained significantly elevated in the tourniquet group.

**Conclusions:**

The findings suggest that RCV offers a more individualized and sensitive approach to assessing perioperative blood loss in TKA. Although conventional methods did not demonstrate significant differences, RCV analysis indicated a more rapid hemostatic response in the non-tourniquet group. Incorporating RCV into routine clinical practice may enhance patient-specific blood management and support earlier postoperative decision making.

## Introduction

Total knee arthroplasty (TKA) is currently the most effective treatment option for pain, restricted mobility, and deformity in advanced-stage knee osteoarthritis, and it remains one of the most performed orthopedic surgeries worldwide [1, 2]. TKA can be performed with or without a tourniquet. The debate over the use of tourniquets in TKA is ongoing, with proponents arguing for their benefits in reducing blood loss and operative time and opponents raising concerns about potential complications and the need for a personalized treatment strategy. Although there is no consensus regarding the superiority of one approach over the other, the importance of a personalized treatment strategy has been emphasized in clinical practice [3].

The impact of tourniquet use during surgery on factors such as blood loss, preoperative and postoperative joint range of motion, complications, pain, and analgesic consumption has been investigated in numerous studies [3–6]. Total blood loss is typically assessed by evaluating intraoperative and postoperative drain output, as well as ongoing tissue seepage, with follow-up conducted using both methods. To assess blood loss, changes in preoperative and postoperative hemogram values ​​are measured or calculated using various alternative formulas, such as the Nadler method, hemoglobin balance, and the Gross method. Although each calculation method includes additional parameters, hemogram values ​​remain the primary variable [6, 7].

The change observed in the individual’s consecutive test results may be due to an improvement or, on the contrary, a deterioration of the clinical condition [8]. However, this change may also be due to natural sources of variation, such as intra-individual biological variation (intra-individual coefficient of variation, CV_I_) and analytical imprecision (analytical coefficient of variation, CV_A_). The reference change value (RCV) concept was developed to account for this inherent variation, encompassing analytical and biological variations [8, 9].

When evaluating sequential test results, it is advisable to use the RCV for tests with an index of individuality (II) less than 0.6; high individuality in these tests suggests a more reliable assessment with RCV [10]. RCV, as an objective tool, has the potential to significantly enhance clinical decision making [9]. Studies have shown that RCV can lead to more accurate evaluations in monitoring side effects, reduce unnecessary retesting, and positively impact treatment durations, thereby accelerating clinical decisions [11–14]. This reduction in unnecessary retesting saves time and resources, making the clinical process more efficient and cost effective. However, to date, no studies have been found in which bleeding is evaluated using RCV, and it appears that this method is not applied to calculate total blood loss during TKA. This study aims to assess whether there is a difference in blood loss between patients undergoing tourniquet-assisted and tourniquet-free arthroplasty using hemogram results evaluated with RCV.

## Materials and methods

### Study design

A retrospective analysis was conducted by reviewing the records of patients who underwent TKA between January 2020 and December 2022. Patients with complete blood count analyses performed preoperatively (Pre-Op), postoperative same day (PO-D0), and postoperative day one (PO-D1) were included in the study. The study was approved by the Kastamonu University Clinical Research Ethics Committee in accordance with the Declaration of Helsinki (approval number: 2024-KAEK-16, date: 07.02.2024). All patient data were obtained from the hospital record system.

The indications for TKA included patients with primary knee osteoarthritis who did not respond to conservative treatments (medications, physical therapy, weight loss, etc.) [15], the presence of severe pain, and Kellgren-Lawrence grade III or IV [16] (characterized by joint space narrowing, subchondral bone cysts, bone sclerosis, and osteophyte formation).

Patients were randomly divided into two groups based on the use of a tourniquet during surgery. The first group (tourniquet group) included patients who underwent TKA using a tourniquet. In contrast, the second group (non-tourniquet group) included patients who had undergone TKA without one.

In the tourniquet group, the tourniquets were applied just prior to the skin incision, in accordance with standard surgical protocols and were deflated before wound closure; the layers were closed in the reverse order of their opening. Intraoperatively, the femoral intramedullary canal entry was not sealed with an autologous bone plug in either group. Drains were used in both groups and removed after 24 h. The same postoperative protocols were applied to both groups for pain management with anti-inflammatory drugs and opioids, antithrombotic and antibiotic prophylaxis agents, and postoperative rehabilitation. Low-molecular-weight heparin treatment was initiated 12 h postoperatively and continued for two weeks. Antibiotic prophylaxis with a first-generation cephalosporin (4 × 1 g) was started one hour before the operation and continued for 24 h [6, 17, 18].

### Data collection and outcome measures

Complete blood counts were performed in the clinical biochemistry laboratory using the Sysmex XN-1000 hematology analyzer (Sysmex Corporation, Kobe, Japan). The analyses included red blood cell count (RBC), hemoglobin concentration (HGB), hematocrit (HCT), platelet count (PLT), white blood cell count (WBC), lymphocyte count (LYM), and neutrophil count (NEUT).

Internal quality control (IQC) results of 2022 were obtained from the laboratory information management system, and the mean CV_A_ values of the hemogram parameters were calculated. Sysmex XN Check (Sysmex Corporation, Kobe, Japan) IQC samples were analyzed at three levels daily. IQC materials with lot numbers QC-13,421,101/02/03 were used in January-February, QC-20,331,101/02/03 in March-April, QC-20,891,101/02/03 in May-June, QC-21,451,101/02/03 in July-August, QC-22,011,101/02/03 in September-October, and QC-22,571,101/02/03 in November-December. IQC results were evaluated based on the Westgard rules and were either accepted or rejected accordingly [19]. The standard deviation (SD) and mean were calculated monthly for the three-level controls of each parameter. The CV for all three-level controls was determined using the formula CV = (SD / Mean) × 100. The monthly CV_A_ values of the tests were derived from the average CV of the three levels. Subsequently, the mean CV_A_ for the twelve months of 2022 was calculated for each test.

The biological variation (BV) data (within-subject biological variation: CV_I_ and between-subject biological variation: CV_G_) were obtained from The European Federation of Clinical Chemistry and Laboratory Medicine (EFLM) BV database [20]. The II for each parameter was calculated using the formula II = CV_I_ / CV_G_ [21]. Total variation (CV_T_) for each test was determined separately using the formula CV_T_ = (CV_A_² + CV_I_²)^1/2^ [10].

Logarithmic reference change factor (RCF) values were calculated using the following formulas based on the logarithmic approach by Lund et al. [22]:

$${\rm{RC}}{{\rm{F}}_{{\rm{up}}}} = \exp \left( {{\rm{Z}} \times {2^{1/2}} \times {\rm{C}}{{\rm{V}}_{\rm{T}}}/100} \right)$$

$$RC{F_{{\rm{down }}}} = 1/RC{F_{{\rm{up }}}}$$

These factor limits were then converted to RCVs to make the results comparable. The distance of the RCF values from 1 was calculated proportionally, and the increasing/decreasing RCVs were expressed as percentages. For example, for the hemoglobin parameter, the RCFup value was 1.07, and the RCFdown value was 0.94. These factor limits were converted to percentages as follows: for upward change, the RCV value was calculated as (1.07–1) / 1 = 7%, and for downward change, it was (1–0.94) / 1 = 6%. In this formula, the Z value was used as 1.65 for a one-sided 95% confidence interval (*p* < 0.05) [23]. The analyses were performed using Microsoft Excel 2013 (Microsoft Corporation, Washington, USA). Changes in hemogram parameters during the early postoperative period (first hour) and late period (the following morning) were evaluated using the calculated RCVs.

### Statistical analysis

Statistical analyses were performed using the Statistical Package for Social Sciences (SPSS) 18.0 (SPSS Inc., Chicago, IL, USA). The normality of the distribution of variables was assessed using analytical methods (Kolmogorov-Smirnov/Shapiro-Wilk tests). Descriptive statistics were reported as mean ± standard deviation for Gaussian-distributed data and median with first and third quartile limits (Q1-Q3) for non-Gaussian-distributed data. The chi-square test was used to determine whether there was a significant difference in gender distribution between the two groups. Since the patients operated with and without a tourniquet formed two independent groups, Student’s t-test was used for normally distributed data, and the Mann-Whitney U test was employed for non-normally distributed data. A p-value of < 0.05 was considered statistically significant.

## Results

The study included 68 patients who underwent arthroplasty with a tourniquet (57 women, 11 men) and 69 patients who underwent arthroplasty without a tourniquet (62 women, 7 men). The ages of the tourniquet and non-tourniquet groups were 68 (ages 63–74) and 67 (ages 63–73), respectively, indicating good homogeneity between the groups in terms of age. There was no significant difference between the two groups regarding gender (*p* = 0.296) and age (*p* = 0.760). The descriptive statistics of the patients’ variables and the preoperative and postoperative late-period complete blood count parameters are presented in Table 1.

**Table 1:** The descriptive statistics of the patients’ variables and the preoperative and postoperative hemogram parameters

	Tourniquet (Pre-op)	Non-Tourniquet (Pre-op)	*P*-value	Tourniquet (PO-D1)	Non-Tourniquet (PO-D1)	*P*-value
Age (years)	68 (63–74)	67 (63–73)	0.760^a^	-	-	-
Female/Male (gender)	57/11	62/7	0.296^b^	-	-	-
WBC (x10^9^/L)	6.6 (5.7–7.9)	6.9 (6.1–7.5)	0.364^a^	9.2 ± 3.1	9.5 ± 2.1	0.538^c^
RBC (x10^12^/L)	4.82 ± 0.51	4.69 ± 0.51	0.138^c^	3.7 ± 0.5	3.7 ± 0.4	0.301^c^
HGB (g/dL)	13.7 ± 1.5	13.1 ± 1.5	**0.035** ^**c**^	10.3 (9.4–11.2)	10.3 (9.9–11.3)	0.265^a^
HCT (%)	41.4 ± 3.9	40.2 ± 4.3	0.094^c^	31.1 ± 4.1	31.9 ± 3.3	0.241^c^
PLT (x10^9^/L)	255 ± 56	259 ± 53	0.600^c^	189 ± 48	204 ± 43	0.068^c^

a: Mann-Whitney U test. b: Chi-Square Test. c: Independent Samples T-Test. WBC: white blood cell count, RBC: red blood cell count, HGB: hemoglobin concentration, HCT: hematocrit, PLT: platelet count, Pre-op: preoperative, PO-D1: postoperative day one

Although there was a statistically significant difference in the preoperative HGB test (*p* = 0.035) between the tourniquet and non-tourniquet surgical groups, there was no significant difference in the HCT (*p* = 0.094) and RBC (*p* = 0.138) parameters. Postoperatively (PO-D1), no statistical difference was found between the two patient groups in terms of the HGB (*p* = 0.265), HCT (*p* = 0.241), and RBC (*p* = 0.301) parameters. The CV of hemogram parameters at three levels, the mean CV_A_ values in 2022, and the desirable CV data are presented in Table 2.

**Table 2:** The analytical imprecision, and desirable CV data of hemogram parameters

Test	CV_level 1_	CV_level 2_	CV_level 3_	CV_A_	CV_desirable_
WBC	1.92	1.57	1.20	1.60	5.5
RBC	0.98	0.97	0.97	0.98	1.4
HGB	1.23	0.85	0.73	0.97	1.4
HCT	1.38	1.25	1.12	1.26	1.4
PLT	5.23	3.55	2.18	3.94	4.5
NEUT	3.45	2.58	2.15	2.81	7.0
LYM	4.80	3.57	3.58	4.06	5.4

WBC: white blood cell count, RBC: red blood cell count, HGB: hemoglobin concentration, HCT: hematocrit, PLT: platelet count, NEUT: neutrophil count, LYM: lymphocyte count, CV: coefficient of variation, CV_A_: analytical imprecision. *Desirable CV data was from the EFLM BV database [20]

The BV data, the II value, analytical imprecision, and the increasing/decreasing RCVs calculated using the logarithmic method for the complete blood count parameters are presented in Table 3. Since the II values for nearly all parameters were 0.6 or below, it was concluded that evaluating serial results using RCV would be more accurate. The highest RCV was found for the NEUT parameter, with 39.6% for an increase and − 28.4% for a decrease. The lowest RCVs were found for the HGB parameter, with 6.9% for an increase and − 6.4% for a decrease.

**Table 3:** Biological and analytical variation data for hemogram parameters along with increasing/decreasing RCVs

	CV_I_	CV_G_	II	CV_A_	RCF_up_	RCF_down_	RCV +	RCV -
WBC	11.1	17.2	0.6	1.60	1.30	0.77	29.7	-22.9
RBC	2.8	7.0	0.4	0.98	1.07	0.93	7.1	-6.7
HGB	2.7	6.2	0.4	0.97	1.07	0.94	6.9	-6.4
HCT	2.8	5.6	0.5	1.26	1.07	0.93	7.4	-6.9
PLT	7.3	16.3	0.4	3.94	1.21	0.83	21.2	-17.5
NEUT	14.1	24.3	0.6	2.81	1.40	0.72	39.6	-28.4
LYM	10.8	22.3	0.5	4.06	1.31	0.77	30.7	-23.5

WBC: white blood cell count, RBC: red blood cell count, HGB: hemoglobin concentration, HCT: hematocrit, PLT: platelet count, NEUT: neutrophil count, LYM: lymphocyte count, CV_I_: within-subject biological variation, CV_G_: between-subject biological variation, II: Individuality index, CV_A_: analytical imprecision, RCF_up_: reference change factor for the upward, RCF_down_: reference change factor for the downward, RCV+: upward reference change value, RCV-: downward reference change value

The percentage changes in complete blood count parameters between preoperative and early postoperative periods, as well as between the early (PO-D0) and late postoperative (PO-D1) periods, are shown Table 4. The results of preoperative and same-day postoperative assessments showed that both WBC and NEUT significantly increased when evaluated using RCV. However, there was no statistical difference between the groups (WBC: *p* = 0.210 and NEUT: *p* = 0.215). RBC, HGB, and HCT significantly decreased in both groups according to the RCV values, but these changes did not result in statistically significant differences (RBC: *p* = 0.156, HGB: *p* = 0.092, and HCT: *p* = 0.052). When the changes between the day of surgery and the first postoperative day blood samples were examined, a median 10% decrease in RBC in the tourniquet group was significant because it exceeded the RCV. In contrast, a 6.5% decrease in the no-tourniquet group, which did not exceed the RCV, was not significant (RCV of RBC: -6.7%). In the late postoperative period (PO-D1), HGB and HCT continued to show significant decreases when evaluated using RCV (HGB in the tourniquet group: -9.9% and in the non-tourniquet group: -7.3%). However, while the HGB parameter showed a statistically significant further decrease, no statistical difference was found for HCT (HGB: *p* = 0.008 and HCT: *p* = 0.152).

**Table 4:** Percentage changes in hemogram parameters over surgical phases

	Pre-op_PO-D0 change, %			PO-D0 _ PO-D1 change, %			RCV %
	with tourniquet	without tourniquet	p	with tourniquet	without tourniquet	p	
WBC	**44.7 (13.4–72.5)**	**30.9 (10.5–48.9)**	0.210^a^	-6.1 (-23.9-22.5)	5.5 (-12.1-21.1)	0.088^a^	+ 29.7
RBC	**-16.2 ± 6.1**	**-14.7 ± 8.2**	0.156^b^	**-10 (-13–5.3)**	-6.5 (-13.4–1.1)	0.095^a^	-6.7
HGB	**-15.9 ± 6.4**	**-14.3 ± 8.0**	0.092^b^	**-9.9 (-13.7–6.4)**	**-7.3 (-12–1.4)**	**0.008** ^**a**^	-6.4
HCT	**-16.1 ± 6.2**	**-14.0 ± 8.1**	0.052^b^	**-10.6 (-13.7–6)**	**-7.4 (-14–3)**	0.152^a^	-6.9
PLT	**-18 (-26–10)**	-14 (-23–7)	0.143^a^	-8 (-16-1)	-5 (-15-3)	0.390^a^	-17.5
NEUT	**88 ± 77**	**73 ± 58**	0.215^b^	-9 (-30-35)	4 (-16-35)	0.235^a^	+ 39.6
LYM	**-25 (-37–1)**	-19 (-29–7)	0.233^a^	-15 (-36-7)	-11(-26-7)	0.364^a^	-23.5

a: Mann-Whitney U test. b: Independent Samples T-Test. WBC: white blood cell count, RBC: red blood cell count, HGB: hemoglobin concentration, HCT: hematocrit, PLT: platelet count, NEUT: neutrophil count, LYM: lymphocyte count, Pre-op: preoperative, PO-D0: postoperative same day, PO-D1: postoperative day one

The early and late postoperative complete blood count results of the tourniquet and non-tourniquet patient groups were evaluated according to the RCV (Table 5). The number of patients showing significant changes according to the RCV for each parameter was categorized for each group. When comparing the groups, a significant difference was found only for the HCT parameter in the pre-op and PO-D0 periods (*p* = 0.036). In the PO-D0 and PO-D1 periods, significant changes were observed in the RBC, HGB, and HCT parameters (RGB: *p* = 0.045, HGB: *p* = 0.009, and HCT: *p* = 0.039).

**Table 5:** Analysis of the number of patients showing significant changes based on RCV for hemogram parameters

	Pre-op_PO-D0			PO-D0 _ PO-D1		
	with tourniquet n (%)	without tourniquet n (%)	p-value^a^	With tourniquet n (%)	without tourniquet n (%)	p-value^a^
WBC	41 (60)	38 (55)	0.536	10 (15)	17 (25)	0.144
RBC	64 (94)	59 (86)	0.096	45 (66)	34 (49)	**0.045**
HGB	63 (93)	59 (86)	0.181	51 (75)	37 (54)	**0.009**
HCT	64 (94)	57 (83)	**0.036**	49 (72)	38 (55)	**0.039**
PLT	35 (52)	27 (39)	0.147	15 (22)	14 (20)	0.800
NEUT	48 (71)	49 (71)	0.956	16 (24)	15 (22)	0.802
LYM	33 (49)	39 (57)	0.349	24 (35)	20 (29)	0.429

a: Chi-Square Test. WBC: white blood cell count, RBC: red blood cell count, HGB: hemoglobin concentration, HCT: hematocrit, PLT: platelet count, NEUT: neutrophil count, LYM: lymphocyte count, Pre-op: preoperative, PO-D0: postoperative same day, PO-D1: postoperative day one

In the pre-op and PO-D0 periods, there was no statistically significant difference between the tourniquet and non-tourniquet groups for RBC and HGB parameters. However, the decrease in HCT was more significant in the tourniquet group. In PO-D0 and PO-D1, the decrease in RBC, HGB, and HCT parameters was significantly greater in the tourniquet group. When evaluated with RCV, the decrease in RBC, HGB, and HCT, indicating more blood loss, was greater in the tourniquet group.

## Discussion

This study examined the changes in the hemogram parameters of patients undergoing TKA with and without tourniquet application. No statistically significant difference in bleeding between the two procedures was revealed, consistent with previous studies [4, 5, 24, 25]. However, due to the high individuality of hemogram parameters (II < 0.6), the results were evaluated using the RCV [19, 26]. To our knowledge, this is the first study to evaluate blood loss using RCV. The findings of this study have significant implications for the management of blood loss in TKA, particularly in understanding the role of tourniquet application and the potential benefits of using RCV for evaluation.

According to the RCV-based evaluation, a significant decrease in bleeding parameters (RBC, HGB, and HCT) at PO-D0 was observed, but there was no statistical difference between the two groups. However, when the change from PO-D0 to PO-D1 was analyzed, the decrease in RBC persisted at a median of 10% in the tourniquet group versus 6.5% in the non-tourniquet group, which is below the RCV threshold of 6.7%. When patients showing significant changes based on RCV were categorized and evaluated, the decrease in 94% of patients was significant only for the HCT test in the tourniquet group on the PO-D0. In the changes between PO-D0 and PO-D1, the decrease in RBC, HGB, and HCT parameters was significantly greater in the tourniquet group. All of these findings indicate that blood loss was greater in the tourniquet group, and hemostasis was achieved earlier in the group without a tourniquet.

Although RCV analysis revealed ongoing bleeding in the tourniquet group on postoperative day one, this finding may appear contradictory to common clinical expectations. However, one possible explanation is the timing of tourniquet release. In our study, the tourniquet was deflated before wound closure, a method that has been associated with increased bleeding according to previous reports by Ishii and Matsuda [27]. Similarly, Rama et al. [28] found that early tourniquet release was linked to higher total blood loss. These findings align with our observations and suggest that blood loss may persist postoperatively in the tourniquet group. This emphasizes the value of RCV in revealing nuanced physiological changes not captured by traditional methods.

It has been acknowledged that population-based reference intervals have limitations in tests with high individuality. In response, RCV has emerged as a powerful tool for evaluating changes in the results of consecutive measurements within an individual, considering both analytical imprecision and intra-individual biological variation [26, 29]. While RCVs may not detect upward changes, they excel at identifying downward changes [29], making them a promising avenue for quickly detecting deterioration in health status, as demonstrated in this study.

Successful patient management after TKA should be evaluated, along with an appropriate blood management strategy [30]. Blood loss during TKA is a significant concern, typically leading to a 2–4 g/dL decrease in hemoglobin concentration [18, 31, 32]. In our study, hemoglobin levels decreased by 2.9 g/dL in the non-tourniquet groups and by 3.4 g/dL in the tourniquet groups. This finding is consistent with the values reported in the aforementioned studies.

Allogeneic blood transfusion is often required after TKA, with rates ranging from 6 to 53% [33]. It has been shown that blood transfusion after knee arthroplasty increases the risk of fluid overload, infection, length of hospital stay, and costs by more than 20% [31, 34]. Additionally, complications related to incompatible transfusions, such as kidney failure, bleeding, and the risk of death, are well known [35]. Therefore, proactive and vigilant management of bleeding is crucial, as it significantly affects the success of knee arthroplasty [31, 35].

In our study, the average preoperative hemoglobin level in both groups was above 13 g/dL, which is known to reduce the need for blood transfusions [24]. We established a trigger level for blood transfusion when hemoglobin fell below 8 g/dL and decided on transfusion based on the clinical and biochemical evaluation (including decreased oxygen saturation in response to increased oxygen demand, decreased lactate levels, tachycardia, and hypotension) [33, 36]. In this study, none of the patients met the criteria for blood transfusion; even in those with hemoglobin concentrations below 8, no transfusions were administered.

Studies have shown that whether a tourniquet is used does not have a statistically significant effect on the amount of bleeding or the need for a blood transfusion [25]. In the studies by Smith et al. [4] and Zhang et al. [5], the use of a tourniquet reduced intraoperative bleeding. However, it was shown that the use of a tourniquet did not provide any additional benefit in terms of postoperative bleeding, total blood loss, or transfusion rates. In our study, when RCV was not included, no statistically significant difference was found between the two groups in terms of total blood loss.

Prasad et al. [35] demonstrated that the lowest hemogram levels were reached on the day after surgery; by day 14, the hemogram levels were almost the same as those on the first day. Another study showed that the blood loss in the first two hours was nearly the same as between 2 and 24 h, constituting approximately 80% of the total blood loss [18]. Consistent with these studies, our study included hemogram parameters from the same day and the following day after the operation to evaluate blood loss management. The assessment of PO-D1 showed that blood loss persisted in the tourniquet group, whereas hemostasis was achieved in the non-tourniquet group.

The shortcomings of population-based reference intervals are well known, with RCV defined as an objective means of indicating an actual change in consecutive patient test results [37]. The role of RCV in the clinical decision-making process has been emphasized as effective [29, 38]. When RCV is included, significant differences in biological variation have been observed in thyroid function [26], kidney function tests, glomerular filtration rate [39, 40], capillary blood glucose [41], amylase, ALT, AST, and ALP [42]. Many tools are being developed to assist clinicians in making accurate decisions; studies have shown high variability among clinicians, especially in clinical assessments, without considering RCV [43]. While RCV has been less successful in detecting upward changes, downward changes are more easily discerned in traditional settings because positive changes significantly increase measurement variance. In contrast, negative changes have been shown to decrease it [29]. Therefore, incorporating RCV into the assessment should be considered a practical approach to evaluating clinical outcomes and treatment strategies, especially in health conditions where bleeding is expected.

### Limitations

This study has several limitations that should be considered when interpreting the findings. First, the retrospective design inherently limits the ability to control for all potential confounding variables, such as differences in surgical technique, intraoperative blood pressure management, or anesthesia protocols, which may have influenced blood loss. Second, although efforts were made to ensure group homogeneity, the absence of randomization may have introduced selection bias. Third, the study relied solely on hemogram-based parameters and the RCV method to estimate blood loss. While this approach introduces a novel and objective dimension, it may not fully capture actual blood loss compared to direct intraoperative measurements or radiolabeled red blood cell methods. Finally, as the study was conducted in a single center, the generalizability of the findings to broader populations or different healthcare settings may be limited. Additionally, surgical duration was not included in the analysis due to the retrospective design and incomplete data in some patient records. However, previous studies—including those by Smith et al. and Davulcu et al. —have demonstrated that surgical time does not have a significant effect on total blood loss in TKA [4, 44]. Future prospective and multi-center studies with larger sample sizes and more comprehensive blood loss measurement methods are warranted to validate our results.

## Conclusion

This original study demonstrates that the RCV, by incorporating both biological and analytical variability, offers a more sensitive and individualized approach to assessing postoperative bleeding in tourniquet-assisted and tourniquet-free total knee arthroplasty. RCV-based monitoring facilitates precise evaluation of hemogram changes, reduces unnecessary testing, enables early identification of hemostatic stabilization, and supports timely clinical decision making, including discharge planning. These findings highlight the potential of RCV as a valuable tool in perioperative blood management, warranting further research into its application across various surgical procedures and its comparison with conventional blood loss estimation methods through prospective studies with extended follow-ups.

## Data Availability

No datasets were generated or analysed during the current study.
